# “The Role of Vitamin D in Cancer Prevention”: Some New Clues on a Fascinating Subject

**DOI:** 10.3390/nu15112560

**Published:** 2023-05-30

**Authors:** Jose M. Martin-Moreno, Alejandro Martin-Gorgojo

**Affiliations:** 1 Department of Preventive Medicine and Public Health, Universitat de Valencia, 46010 Valencia, Spain; 2 Biomedical Research Institute INCLIVA, Hospital Clínico Universitario de Valencia, 46010 Valencia, Spain; 3 Madrid City Council, STI/Dermatology Department, 28006 Madrid, Spain; alejandromartingorgojo@aedv.es

Increasing evidence from experimental animal nutrition studies suggests that vitamin D may potentially influence apoptosis and tumor-associated angiogenesis, reduce the initiation of carcinogenesis, and delay the multiplication and proliferation of tumor cells. In humans, it is known that the incidence and mortality rates of some malignant tumors are lower in individuals living in southern countries, where exposure to ultraviolet light from the sun and to vitamin D itself is more intense. Additionally, although these findings derived from ecological studies do not necessarily prove causality, the excitement about their hopeful implications for the prevention of malignant neoplasms has been partly fueled by the publication of various observational epidemiological studies and a limited number of intervention studies, which have offered some evidence in support of this claim. Unfortunately, the results so far have not been thoroughly consistent. Overall, evidence is not solid or comprehensive enough to firmly establish that taking vitamin D can prevent cancer, while, in a complementary manner, the possibility of its associated beneficial effects has not been ruled out either. The present *Nutrients* Special Issue entitled “The Role of Vitamin D in Cancer Prevention” includes seven articles from different research groups, which are briefly summarized below.

The article by Dr. Moisejenko-Goluboviča and colleagues [1] discusses the relationship between vitamin D and basal cell carcinoma (BCC), the most common type of skin cancer, which is primarily caused by ultraviolet radiation. The study found that most BCC patients were deficient in vitamin D and that low vitamin D levels were associated with aggressive and recurrent BCC in susceptible male individuals. The study also observed a link between vitamin D and the proteins involved in its metabolism, such as vitamin D binding protein (DBP) and sonic hedgehog protein (SHH), suggesting a potential protective effect of vitamin D on cutaneous neoplasms. 

The manuscript by Dr. Sánchez-Bayona et al. [2] explores the association between vitamin D intake and the risk of obesity-related cancers. The study population included 18,017 participants from the SUN Project, a Spanish cohort of university graduates, with a median follow-up of 12 years. In this middle-aged Spanish population, no significant association between vitamin D intake and obesity-related cancer risk was found after adjusting for potential confounders, whereas the authors also acknowledged that the individuals in the cohort were relatively youthful and physically fit, with a mean body mass index that predominantly fell within the normal weight range. These traits could also help to clarify the lower occurrence of cancer observed in this specific cohort. 

Dr. Chartron et al. present the results of a phase II trial [3] evaluating the safety and efficacy of high-dose oral vitamin D supplementation in 44 patients with early breast cancer receiving adjuvant chemotherapy; it was deemed safe and effective in correcting vitamin D deficiency in these patients.

The paper by Dr. Brenner and colleagues [4] discusses the potentially protective effects of vitamin D_3_ supplementation against cancer mortality, particularly in relation to body mass index. The VITAL study, which included over 25,000 participants, found that vitamin D_3_ supplementation was effective in reducing cancer incidence and advanced cancer for normal-weight participants, but this impact did not occur in overweight or obese participants. To understand this variation, the researchers conducted complementary analyses of published data from the VITAL study and found that normal-weight participants in the control group had a higher risk of any cancer and advanced cancer compared to obese participants. The authors attempt to explain this paradox by suggesting that perhaps the relatively short follow-up period of the VITAL study (median 5.3 years, with a range of 3.8 to 6.1 years) may explain this apparently unexpected observation. Since most cancers take several years to manifest clinically, this could explain the observed findings in this group, whereas no such patterns were seen in the intervention group, suggesting that pre-diagnostic weight loss in cancer patients and the preventive effects of vitamin D_3_ supplementation against cancer progression could be plausible explanations for the body mass index–intervention interactions, and conclude that their analysis “supports suggestions of a major protective effect of vitamin D_3_ supplementation against cancer progression, either from pre-clinical to clinical cancer or after clinical manifestation.”

Dr. Henn et al. [5] review the relationship between vitamin D status and cancer risk, and the potential role of cholecalciferol supplementation in cancer prevention. While intensive analyses have identified biological mechanisms that may explain the association between low vitamin D status and increased cancer risk, large randomized clinical trials have not yet shown a clear benefit of cholecalciferol supplementation in cancer prevention. Attempting to reconcile these findings by reviewing the existing literature, the authors identify serious limitations in the design and analysis of the studies carried out to date, and propose criteria for interpreting study results. In this way, and while acknowledging interindividual variability in response to cholecalciferol supplementation and promoting customized interventions that foster a healthy environment and responsible health behaviors, they also provide valuable insight in this paper in the form of an exhaustive evaluation of the criteria currently used to interpret findings, suggesting other criteria that may enable us to establish reliable conclusions in this area.

The article by Dr. Palanca and her team [6] debates the controversy surrounding the role of vitamin D in thyroid cancer. While previous studies suggested that high levels of serum vitamin D protect against thyroid cancer, recent data show that circulating vitamin D concentration is inversely correlated with disease aggressiveness and poor prognosis, but not necessarily tumor initiation. Despite the mixed evidence, a growing body of research supports the potential anti-tumorigenic role of vitamin D and its usefulness as a secondary chemopreventive agent. The article also features recent findings on the association of vitamin D status with thyroid cancer risk, prognosis, potential mechanisms, and chemopreventive utility. 

Finally, the manuscript by Dr. Martin-Gorgojo et al. [7] reviews the controversies and conflicting epidemiological evidence regarding the relationship between vitamin D and non-melanoma skin cancer and cutaneous melanoma that are possibly a result of the confounding effects of sun exposure and other factors. Three practical recommendations are set out: (1) individuals at risk or with a personal history of skin cancer should continue to follow sun protection recommendations, (2) vitamin D should preferably be sourced through diet, and (3) serum vitamin D checks are recommended for patients with melanoma or at risk of cutaneous cancer to detect and avoid insufficiency. 

We consider these seven contributions to be of great relevance and are confident that readers will find interesting and practical insights in these manuscripts. At the same time, it is evident that there is a need to generate more research to clear up the unknowns and to obtain better answers and practical actions related to this fascinating subject. It is a noble endeavor certainly worth our efforts. 
